# Blockchain as an Approach for Secure Data Storage on Digital Consulting Platforms

**DOI:** 10.1007/978-3-030-53914-6_6

**Published:** 2020-06-25

**Authors:** Sebastian Gerth, Lars Heim

**Affiliations:** 1 Hanze University of Applied Sciences, International Business School, Groningen, The Netherlands; 2 Loughborough University, School of Business and Economics, Loughborough, Leicestershire UK; 3 Nordhausen University of Applied Sciences, Chair of Digital Management, Nordhausen, Germany; 4 University of Erfurt, Thuringian Competence Center Economy 4.0, Erfurt, Germany; 5 Clausthal University of Technology, Clausthal-Zellerfeld, Germany

## Abstract

This chapter examines the concept of data security in a society increasingly shaped by digital technologies. We show how secure data storage can be optimised regarding digital documentation in the implementation of health-related service offers based on established procedures. Security and privacy of data are therefore particularly important in this subject area since highly sensitive data is stored and processed during health-related online consultations. The advent of blockchain technology provides a valuable opportunity to create trust in digital platforms. After relevant concepts and terms have been clarified, the functionality of the blockchain in general, as well as the different types, will be discussed. From this, options for the use of online consulting are developed and illustrated on the basis of three use cases.

## The Relevance of Digital Consulting Platforms for Entrepreneurs Considering Blockchain Technology

Due to its decentralised mode of operation, blockchain technology enables data to be stored more securely than the centralised methods of data storage that have been widely used up to now. Existing uncertainties have been publicly demonstrated by recent data scandals: For example, the so-called Doxing Gate at the end of 2018, in which the online user “0rbit” or “G0d” made celebrities’ data publicly accessible as in a kind of advent calendar. Just as well known is the Cambridge Analytica scandal in 2016, when millions of Facebook data were illegally evaluated for Donald Trump’s election campaign (Gerth and Heim 2020). These incidents show that we live more in an age of trust than in an information age. While information on electronic news, social media and knowledge platforms is continuously available and is exponentially growing in volume (Demary 2016; Jaekel 2017; de Reuver et al. 2018; Zehir et al. 2020), trust is a commodity that the players must either first strategically acquire or laboriously recapture when they hope to gain the favour of the users for digital services such as digital consulting platforms (DIVSI 2017a, b; Diekhöner 2018).

The range of *digital services* is extremely diverse and extends from the (partially) public provision of information or communication options such as chats, e-mail or similar, to online banking, billing and payment systems, for example, in the case of e-commerce solutions, to e-learning and concrete personal advisory services (Hanekop et al. 2001; Bruhn and Hadwich 2017; Stich et al. 2019). The borders between services are often blurred, since social media platforms, for example, allow multimedia communication between at least two parties, money transfer, discussions in forums and so on. However, all digital services generally have in common that they are provided by centralised institutions, which themselves have a high degree of digitisation and are represented via digital platforms (Jaekel 2017; Kofler 2018). As a result, the business models are highly scalable, and corresponding organisations can have considerable market power (Gundlach 2009; Täuscher et al. 2017). Thus, in this chapter, a digital service is understood to be a service offered on an online platform to solve a socially or individually relevant problem, in the course of the use of which personal data is collected, stored and processed by the offering institution. As already indicated, the collection of personal data demands a certain level of data security. This is where blockchain technology can provide a remedy.

There is still disagreement in the scientific literature about a generally valid definition of blockchain, as different scientific directions, such as economics, computer science and law, meet and deal in parallel with the common terms used in the practical application of the technology (Gerth and Heim 2020).^1^ In a comprehensive, interdisciplinary analysis, Meijer (2017) summarises all relevant definition components from the scientific, but also from the application-oriented literature. This results in the following definition, which is used in this chapter:

‘Blockchain technology is a distributed, shared, encrypted, chronological, irreversible and incorruptible database and computing system (public/private) with a consensus mechanism (permissioned/permissionless), that adds value by enabling direct interactions between users’ (Meijer 2017, p. 39).

In other words, a blockchain is a digital accounting system in which several actors—first and foremost transmitters, receivers and operators (of the nodes) of the decentralised network—are involved (Burgwinkel 2016; Drescher 2017).

In the following, this chapter aims to highlight the contribution of blockchain technology in creating trust in digital service and consulting offerings through data security.

## Underlying Concepts: Data Security and Data Protection in Online Consulting and Blockchain Technology

*Online consulting*, as a specific form of digital services, can be described as an exchange of information between at least two parties via digital channels based on natural and/or artificial intelligence. On the level of content, the counterpart takes care of a (e.g. physical) problem of one or more clients individually in order to improve the (e.g. health) state. Such a consultative institution can be a human being on the one hand, and a digital counterpart, such as an artificial intelligence in the form of an algorithm (e.g. a bot), on the other.

### Data Security and Data Protection

The handling of data in communication and storage, especially against the background of individual problems, is highly relevant. Discretion can, for example, be ensured by a self-imposed duty of confidentiality, the existence of which and the mandatory compliance with which should be publicly communicated. Ultimately, this is a way of establishing anonymity towards third parties. It appears useful if those seeking help always have the same contact person, although complete digital documentation in the form of a customer administration—for example, by means of a personalised e-filing system (also known as EHR systems, electronic health records; Ströher and Honekamp 2011; Karg 2013)—provides the possibility that colleagues can also offer their help in an emergency. Furthermore, several consultations are often necessary to solve a problem and a future request for help can be based on the solution history of the respective client. Availability can be controlled via cloud applications and the allocation of appropriate access rights to the personal e-file. While this dimension focuses on the management of an organisation, the protection against manipulation, disclosure and loss of relevant data mainly concerns the underlying IT infrastructure. Privacy is an essential umbrella for both aspects: on the one hand regarding the consultant/intermediary–client relationship, and on the other hand of course regarding to data security and data protection (Grimm and Bräunlich 2015). While *data security* should protect data, *privacy* protects people. Data security concerns the protection of data against abuse, falsification and loss or non-availability. Data protection concerns the use of personal data by authorised persons. Data protection is primarily of interest from the perspective of the data subject, while data security is primarily considered from the perspective of the data processor and owner (Bühler et al. 2019). Data security is thus aimed at IT systems and therefore at the technical component of digital services, while data protection refers to stored content and hence the legal component. The latter is usually regulated by specific directives such as the European Data Protection Regulation (GDPR) and must be implemented by intermediaries or organisations involved in online consulting. The former, however, requires consideration because of the relevance of blockchain technology for digital consulting platforms.

In order to securely archive long-term data in digital form (Hackel and Roßnagel 2008), it is possible to work with local systems, i.e. software installed on local computers and/or storage on individual data carriers. Modern working environments, on the other hand, use certain cloud systems as a de facto standard. The advantage is, above all, the ability to work independently of time, location and device, as well as collaborative work due to the constant availability of the owing to its storage on servers that are usually provided externally. These are usually operated in computer centres, which in turn are specialised in their operation, administration, security and access protection as a business model. Hardware acquisition and maintenance are therefore no longer necessary if external services are used; the services provided can be easily adapted to the organisational development and, if necessary, several existing or new company locations can be easily integrated; SaaS models for, for example, specific CRM systems for documenting customer contacts also allow reliable cost calculation based on monthly invoices. The only requirement for its use is sufficiently fast Internet access. Employees are then given access to the files relevant to their work, which can sometimes also be edited collectively.

The points mentioned above already show that not only clients must have confidence in the provider in order to use it, but also the management of the organisation itself must trust in cloud providers with regard to data security, sovereignty, access and processing as well as storage location, maintenance, failure protection and so on (Walterbusch and Teuteberg 2012; Buch et al. 2014; Backhaus and Thüring 2015), which provide and ensure the technological basis for the work on the client. In addition, dependence on the cloud or SaaS provider also has a significant impact, as non-compliance with data protection and security standards ultimately falls back on the institution. This can not only result in image problems but also sometimes lead to immense downtime costs in the event of the cloud provider’s insolvency.

The current practice of data processing and the reasons mentioned above motivate entrepreneurs and their teams, as same as individuals to think about alternatives and/or possible solutions. Trust in centralised systems can be created, for example, through anonymisation (e.g. through onion routing, as in the TOR service), encryption technologies (Schulz 2016; Petrlic 2017), digital signatures (Kumbruck 2000; Bertsch 2002), VPN connections and/or legally and audit-proof archiving (Hackel and Roßnagel 2008). In addition to these instruments, the blockchain also serves to increase not only data security, but also data protection, as described in the following section.

### Foundations, Advantages and Disadvantages of Blockchain Technology

**Fig. 1:**
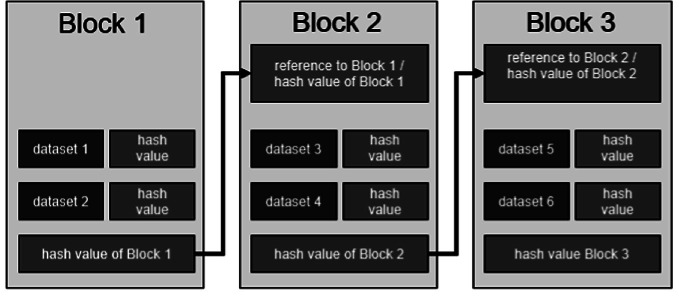
Illustration of the block generation of a blockchain. *Source* Own illustration according to Burgwinkel (2016)

The technological basis of a blockchain is formed by the so-called data blocks: each block contains at least one data record (e.g. digitally recorded contents of a consultation), a timestamp (date and time of the conversation), transaction data (in the form of addresses of the parties involved, e.g. from consultant to client) and a cryptographically secure, so-called hash value of the previous block as well as the verification sum of the entire blockchain. The hash value is a character string of a certain length that acts as a check value: the blocks that build on each other are cryptographically linked using the hashes to form a chain (e.g. to map the course of a consultation over a longer period of time). This is where the name of the technology is derived from (Swan 2015; Mougayar and Buterin 2016) (Fig. 1).

The entirety of these signed and sealed blocks is called a blockchain. It is stored on several network computers or nodes; thus, it is decentralised and hence a neutral system of information processing (Burgwinkel 2016). To participate in the blockchain, a software access, the so-called wallet, is required. Access is gained via digital keys: the public key is comparable to the international bank account number (IBAN) known from the banking sector, and the private key is like the secret personal identification number (PIN). The public key can, therefore, be easily communicated to third parties as an address for transactions, while the private key serves as an access password to the wallet and for transaction verification: in order for the participants in the public blockchain to agree on an identical version of the same block, a consensus must be reached—for this purpose, there are various mechanisms for signing or creating blocks.^2^ This process is called mining. Those actors who are involved in this process are called miners—in the above metaphor, these are, so to speak, the accountants of the blockchain.

The *advantages* offered by blockchain are numerous. First, the technology creates a new level of transparency, as all transactions can be monitored. Furthermore, the code of the blockchain is often freely available. Decentralisation ensures that each participant (e.g. consultants and clients involved in the network) has equal rights and always a synchronised, validated and up-to-date version of the blockchain. This also means that decisions (e.g. on code updates) must be made by a majority. By storing the blocks in the distributed network, the verification of the transactions by numerous nodes as well as the cryptographic encryption and the complex consensus mechanisms, a high degree of integrity and manipulation security is generated in a blockchain (Hooper 2018). This makes it highly reliable and trustworthy (e.g. for verification to a third party, such as a health insurance company).

Decentralised data processing with many replications also leads to a high degree of reliability (Burgwinkel 2016). This redundancy is thus effective protection against attacks and data loss. Furthermore, the interlinking of the individual data blocks with the help of transparent hashing in the distributed network ensures good traceability of the permanently traceable transaction history (e.g. in the form of a medical history; Consultancy UK 2017). The blockchain also enables transactions to be processed faster and more efficiently than previous methods. This can lead to an increase in quality at lower costs compared to other IT systems. The technology also ensures disintermediation, i.e. the streamlining of value chains, which can prevent dominant market positions (Song et al. 2016). Particularly, (fee-based) intermediary players are affected, which could be eliminated by using a blockchain (Düring and Fisbeck 2017).^3^

However, the advantages are also countered by several *disadvantages* (Cap 2019; Kossow 2019). Currently, the scaling of blockchains is problematic: since each node of the network has to store all data, large amounts of data of several terabytes can accumulate in a short time, especially with numerous transactions (e.g. payments), as the blockchain increases in size with each block. It is therefore difficult for many new users to join a blockchain at short notice. With the current broadband and storage capacities, participation is therefore difficult, especially for private individuals, or involves great demands on the technical infrastructure. Ultimately, this also limits the lifetime of a blockchain if the expansion of memory and network speed is lagging behind the resource requirements of a blockchain, and thus successive nodes will disappear, since for instance the expensive hardware is no longer affordable.

Concerning the transactions, there are two noticeable features in particular: on the one hand, the actual transaction must also be signed and synchronised, which is why a blockchain has a significantly lower performance about the speed of the transactions compared to a classic database, which only stores the final state and not the entire transaction history as does a blockchain. It should be mentioned, however, that the difficulty of capacity and confirmation delays is an increasingly less weighty argument against the blockchain, mainly due to the lighting network.^4^ On the other hand, revising transactions is virtually impossible—the stability postulated above as an advantage is thus also a weakness. This applies in particular to public-permissionless blockchains (see the following subchapter, in particular, Table 1); in blockchains with a restricted consensus, this may be possible because the group of validators is clearly defined and they can decide on a rollback by majority vote (Baumann et al. 2017). The above-mentioned advantageous transparency ensures, especially in the case of public blockchains, that everyone can use the public key to view the transaction history—i.e. income, expenses and prices—via an explorer, which is not desirable for every potential participant and is therefore disadvantageous at the same time. This is particularly relevant for automated big data analyses, for example, regarding systematics of transaction flows. An additional problem is access to the blockchain: if a user loses the private key required for validation, he also loses irretrievable access to his wallet and thus to the blockchain (Dasu et al. 2018). While this problem mainly affects individual participants, another problem arises on a collective level: if the actors involved in a blockchain do not have a consensus on the future of a blockchain project since the majority decision process can sometimes be difficult for actors unknown to each other due to a lack of trust (which is why majority voting is both an advantage and a disadvantage), it can lead to the so-called forks, i.e. to splits and thus fragmentation of one and the same blockchain. This can lead to uncertainty among users, as they may then no longer know which blockchain is the one with the more promising future. Another difficulty can be the integration of a blockchain into existing IT infrastructures. This applies equally to hardware and software at the technical level and change management at the employee level.

**Table 1:** Types of blockchain technology

Dimension		Validation		
		Permissionless	Permissioned	
			Single organisation (single authority blockchain)	Consortium (federated blockchain)
**Access**	**Public**	Public-permissionless	Public-permissioned	Public-permissioned
	**Private**	Private-permissionless	Private-permissioned	Private-permissioned

*Source* Own illustration

## Blockchain Typology and Its Usage for Consulting Platforms

The use of blockchain technology in connection with online consulting is often discussed in the medical field (Hölbl et al. 2018; Agbo et al. 2019). However, from the advantages and disadvantages explained in the previous section, it is clear that not all kinds of blockchains are suitable for documenting data of digital consulting platforms in a data protection-compliant manner and thus sufficiently protecting privacy, as these data would be visible to every participant of the blockchain (Pesch and Böhme 2017; Bogdan 2018). Nevertheless, one can distinguish between different blockchain types which offer different application possibilities due to their different configuration (Meijer 2017; Meier and Stormer 2018). According to Kudra (2018), two essential dimensions are used for this purpose:

*The* ‘*access*’ *dimension*: User rights regarding read and write rights and the execution of transactions (public vs. private)

*The* ‘*validation*’ *dimension*: User rights regarding participation in the consensus mechanism (permissionless vs. permissioned)

These two dimensions can be combined, allowing four blockchain types to be distinguished and defined (BitFury Group 2015; Kravchenko 2016; Meijer 2017; Carson et al. 2018; Kudra 2018). These are summarised in the following table.

Public blockchains are therefore intended more for the use by individuals: they retain control over their personal data and can carry out transactions of various data, such as information, financial resources, etc., quickly and cost-effectively without being dependent on a central agency. Private or federated blockchains are predestined for private companies and externally segregated, closed groups, which have to limit the activities in their network to a certain group of people. They offer the efficiency and transparency of blockchain technology in a protected environment that cannot be seen by outsiders. On a private blockchain, access to it—for example, through a digital consultation request with subsequent consultation—is approved by the operator or consultant and thus ultimately by a specific institution. At the same time, it can be defined within the organisational structure which employee can perform specific tasks based on the stored data. For example, a consultant needs information on content, while the payment for the service used is mainly of interest to the finance department.

An institution using a private blockchain retains complete control of the system because all users and all operators of the consensus mechanism are known. In contrast to a public blockchain, trust in the validators is therefore necessary (Buterin 2015). Another significant difference is the reduced transparency: the code of the blockchain cannot usually be seen by the users (Wüst and Gervais 2017). External parties—such as health insurance companies, the employer, or friends and acquaintances of the person seeking help—cannot access the system either. On the one hand, this serves to protect the blockchain and the data it contains, but it also prevents the blockchain from being developed further by a majority of users. The question in each individual case is always whether this is necessary. Just like the validation of transactions, the further development and updating of the blockchain falls to the limited group of validators (Baumann et al. 2017). According to Buterin (2015), an extension or improvement of the blockchain is thus much easier to achieve, since, for example, reconciliation processes can be streamlined. The revision of transactions is also possible in this environment through a rollback, since the group of validators is clearly defined (Baumann et al. 2017). This may be necessary if transactions have been incorrectly validated, e.g. if software errors or attacks have resulted in incorrect prices for the services provided.

Private blockchains use different types of consensus mechanisms than public blockchains, which can validate at a much higher speed than, for example, the above-mentioned proof-based consensus mechanisms (Wüst and Gervais 2017). Particularly noteworthy here are the Byzantine fault tolerance (BFT) based consensus protocols, such as the pBFT (Wüst and Gervais 2017; Castro and Liskov 1999). This is mainly due to the fact that only a few, very powerful network nodes are required for validation: both data transfer rate and loading time are significantly faster with these consensus mechanisms.^5^ The resilience of this blockchain type against hacker attacks, data loss and system failures is much higher when compared since they store data only on a selected set of computing systems (Baumann et al. 2017). Private blockchains are also very scalable and can be easily extended if necessary. Therefore, it is also well possible to test them initially on a small scale and if successful in expanding them (Carson et al. 2018). Legal framework conditions can also be clearly defined, as the blockchain can be unambiguously assigned to a company or another user group (Bogensperger and Hinterstocker 2018). These aspects speak in favour of using private blockchains when setting up digital consulting platforms; however, their centralisation is problematic on top of the disadvantages mentioned above.

In order to break up the centralisation of private blockchains, the so-called federated blockchain can be considered as an extension of the latter (Gerth and Heim 2020). In such a case, more than one institution is responsible for the maintenance of the network or validation. This results in mutual control since the majority of the institutions makes decisions for the benefit of the network. Accordingly, such a consortium reaches a consensus if the majority votes for a certain action (e.g. a change of code, access rights, etc.). Wrong decisions or manipulations by individuals can thus be prevented as far as possible, and the advantages of (limited) decentralization can still be used. In case of establishing online consulting platforms, such a regulative acting consortium should accordingly be composed of experts who are involved in the added value of online consulting: representatives from civil society, professional associations and professional federations and ultimately, for example, market active companies, medical and/or psychological associations and/or health insurance companies, provided that they contribute a share of the financing. The blockchain then serves as a digital instrument that creates trust between all parties involved in the consulting process and at the same time enables the progress of this technology.

## Case Studies: Telemedicine, Patientory and Medblock

Practical implementations of digital consulting platforms based on the blockchain technology are still rare due to the novelty of the technology and its areas of application. In the following sections, three relevant cases will be presented.

### Telemedicine—COVID-19-Pandemic

One possible use of blockchain-based digital consulting platforms in the context of the healthcare system and healthcare provision is the remote diagnosis of disease symptoms by medical personnel. The relevance of such services seems to be high, especially against the background of the COVID-19-pandemic (Khan et al. 2020). Due to the exponential, worldwide spread of the disease and its typical symptoms, which are similar to those of common influenza, the short-term need for diagnoses increased rapidly. In order to reduce the risk of infection for other patients and the medical staff involved, a remote diagnosis option appears to be highly appropriate.

Such a service not only helps to counteract panic but also to collect valuable data that can be used to contain the disease. A system of this kind also makes sense because of the containment measures that came with the COVID-19-pandemic, such as the quarantine of individuals and curfews that were imposed in many places. It could not only assist in the initial diagnosis, but also serve as a basis for further monitoring of quarantined patients. Especially for efforts to contain such epidemics and pandemics, early detection and, as a result, the quality of the data obtained is important (Williams et al. 2013). In addition, patients must be certain of the anonymity and purpose of their patient data and the qualifications of the medical staff treating them. A solution based on a federated blockchain, as described above, could guarantee data quality and security. Accordingly, not only patients would benefit, but also forecasts with higher reliability could be made. A consortium that could oversee the consensus mechanism of such a blockchain could consist of government institutions, hospitals and family physicians. The authors are not aware of any telemedicine company that already relies on the blockchain technology.

The Swedish start-up Kry (kry.de), however, is pursuing an approach that moves in this direction (Blix and Jeansson 2018). Founded in 2015, the company first tested the marketability of telemedicine in Sweden. It entered the German market in December 2019, prompting patients to book a video consultation hour in the smartphone app and answer a few questions about their complaints (Stübner 2020). Such questions are intended to help the doctor prepare for the video chat. At the latest, after 20--30 min—as promised by the start-up—a doctor will get in touch. The company works together with physicians who can issue a prescription, a sick note, or a referral to a specialist. The company also cooperates with the online pharmacy DocMorris, which can deliver the required medication to the patient on prescription if required (Stübner 2020). Although the company is not yet using blockchain technology at the time this text was written, such technology could be used in the future. This could, for example, also make the issuing of prescriptions via the service to patients more secure. During the COVID-19-pandemic, Kry used its existing infrastructure to provide free video consultations to patients with COVID-19-symptoms (KRY 2020).

### Patientory

Patientory (patientory.com) is one of the first providers of distributed apps (dApps) and blockchain-based software solutions for the healthcare industry, which meet the complex challenges of the healthcare sector (Warner 2019). The purpose of such apps is to enable consumers to better manage their own health information and thereby improve their quality of life and health while providing benefits to all stakeholders in the healthcare industry. In order to offer such solutions, the PTOYNet blockchain was launched and additionally, the Patientory Association was founded in 2017 as a global non-profit organisation consisting of institutions of the healthcare industry. The members of the Patientory Association form the consortium to monitor the consensus mechanism of the federated PTOYNet blockchain (Patientory Association 2019).

On the one hand, patientory’s dashboard software provides institutions, service providers and insurance companies with a simple and secure means of storing and better managing health information. It thus serves the health management of a population by regulating and protecting the patient data in the blockchain while providing easy, secure access to actionable health information and administrative decision support. It also enables physician-coordinated patient care that can be fully managed through the platform. On the other hand, the mobile app enables consumers to efficiently track and manage information about their own health and any costs associated with it. Furthermore, the secure transfer of the users’ medical information, which is secured within the PTOYNet blockchain, is facilitated. With these services and benefits, patientory seeks to revolutionise the relationship between patients, physicians and healthcare institutions by utilising blockchain technology (Patientory 2018). It is critical to note that there is still no generally recognised blockchain for the healthcare sector. Nevertheless, patientory has taken an important step in this direction by founding the Patientory Association as a supervisory consortium.

### Medblock

The London-based company MedBlock (medblock.co.uk) intends to store patient data using blockchain and integrate it into the medical treatment process. The company is focusing on a b2b business model which not only allows secure and decentralised data storage and exchange but also wants to enable (predictive) analyses of the available data. The company connects existing EHR systems onto the blockchain network enabling the automation of arbitrary business processes using the data. It, however, does not explain the exact process of data analysis. This is particularly regrettable because the use of artificial intelligence should certainly offer added value here, while the communication between the blockchain and the algorithm would be worth discussing. Regardless of the procedure, the evaluation of the data should always be carried out under the control of a physician who is supported by modern technology.

Particularly against the background of increasing globalisation, it seems to make sense to store health data securely and at any time worldwide for quick comparison during treatments. This is especially true for emergencies, as rapid data availability can be crucial for targeted and successful treatment. For the patient, a transparent insight into the data is relevant for an overview of their own health and correct billing. They are informed about updates on the blockchain by e-mail. Doctors can avoid multiple documentation of the same diseases and thus bureaucratic effort by updating existing entries. Insurance companies can make data retrieval more efficient and coordinate any difficulties with all parties involved on the same data basis.

MedBlock promises to connect existing electronic health record (EHR) systems with the blockchain, without specifically addressing the technical implementation of the interfaces. The company relies on a private blockchain and uses technologies from IBM Bluemix, the IBM Cloud and IBM Watson Health. Against the background of the points discussed above, this seems to be the best solution so far. According to the company’s information, the blockchain is based on the Hyperledger Fabric v.1.4 platform (as of April 2020), as the following figure shows. MedBlock cooperates with Altoros (Sunnyvale, CA, USA) on the development side.

It should be critically noted that there is not yet a standard for storing patient data in the blockchain and that this can probably only be developed by a consortium of relevant, globally active players in the health industry or even the WHO. In this case, a federated blockchain would have to be used. It is therefore fundamentally questionable whether individual companies without certification—it may be that MedBlock will receive one in the future—will achieve a high reach in the field of healthcare and blockchain.

Given the disadvantages of a blockchain, as discussed above, it is debatable why the business model should not be implementable with a cloud-based, encrypted database; this would simplify the desired data analysis in particular, but would possibly be less performant. It is also questionable what happens to patient data if the private keys are lost. MedBlock itself provides a solution by enabling patient-side authentication via fingerprint. The combination of data analysis—whether blockchain-based or not—and treatment methods seems particularly interesting: evaluated data could possibly also contain information on more precise (surgical) procedures that robots would be able to perform much better than humans. A major advantage of a blockchain-based solution is that intermediaries are no longer necessary, thereby strengthening the direct doctor--patient relationship. All in all, the company appears to be still at the beginning of its business activities, which is not unusual given the novelty of the technologies and the associated problems to be solved.

## Conclusion

The topic of digital services has already been established in social and scientific discourse for several years, and the blockchain technology is increasingly gaining profound interdisciplinary attention. The growth in interest in the blockchain technology is shown for instance by the worldwide patent applications with blockchain reference per year, which are rapidly increasing since 2013 and have tripled from 2017 to 2018 (IPlytics 2019). So far, however, a linking consideration of these two technological currents has been largely overlooked. This chapter counters this desideratum by highlighting relevant terms using the example of online consultations, especially in the healthcare sector and the possibilities of creating and maintaining data security through the blockchain. This new technology makes it possible for the first time in history to increase the security level through a technically (or at least organisationally) decentralised solution, as security no longer needs to be centrally placed in an institution (Wildhaber 2016; Tapscott and Tapscott 2018). Transparent end-client communication regarding the functionality of the system, the technical background, and the various user groups that have access to the data are essential in this context. Most importantly, the system must be easily accessible for a user, trivially usable (UX) and highly trustworthy. Ultimately, it is particularly the users who are strengthened by the inclusion of a blockchain in digital services: they are given more control over their personal data and the transactions themselves and thus over their own privacy—provided they understand what opportunities and risks the technology as a whole brings with it (Bogdan 2018). This is also reflected in the three cases presented. The users of patientory, for example, get increased control over their health data. In the same way, the users of MedBlock and of Kry or other telemedicine providers are also strengthened—for example, by the gain in flexibility and confidence in their data security.

Users are currently accustomed to centralised control systems in various areas of social life. This is mainly because responsibilities can be assigned directly. With a blockchain, this is not the case. Participants should, therefore, always be aware that the risk ultimately lies with each user himself or herself. Conversely, this means that, especially in a transitional period, online consulting organisations must ensure that users can make use of both blockchain-based and “traditional” forms of data processing. On the platform side, the question of integrating previous data processing into the blockchain arises. Furthermore, questions of digital ethics (Capurro 2017; Grimm et al. 2019) will also have to be discussed—especially regarding the unavoidable permanence of the information stored on the blockchain. Governments should also create legal models and instruments to provide a legal framework for the management of digital assets. Regulatory supervision and thus also centralisation, however, are controversial, as it takes the idea of decentralisation, which underlies the blockchain technology, to absurdity.

Overall, it can be stated that private (and sometimes federated) blockchains appear to be particularly suitable for data processing in digital services by companies: these types of blockchains combine all the following advantages which allow those seeking and receiving help to interact with each other as best as possible without the risk of data being leaked to third parties: integrity, manipulation and failure safety through transparency, decentralisation, majority principle and cryptography.

## Footnotes

1 Blockchain technology is a comparatively young technology, which has been used mainly for online or open access publications to date. This is reflected in the consequent selection of sources.

2 *Proof of work* (PoW) means that the miners must prove that they have made a certain amount of effort in the verification process. The idea is that the miner demonstrates a conscious willingness to actively participate in the blockchain by using his own energy—i.e. electricity to operate one or more computers, as well as time and equipment. In the PoW, numerous arithmetic operations are performed to create a chain of predefined length from a chain of characters of any length using mathematical hash functions. In this way, the legitimacy of the transaction is checked using resources. The first miner to find the solution is then rewarded (e.g. with a certain value of a cryptocurrency). In the case of PoW, the blockchain is vulnerable to the so-called 51% attacks: If an actor succeeds in controlling 51% of the nodes or computing power for mining, the transaction history can be manipulated—for example, by using the same money several times for purchases, e.g. of Bitcoin. This is not possible with the proof of stake methodology. In *proof of stake* (PoS), the blockchain network itself uses a weighted random selection to reach a consensus on who can create a new block. Moderating aspects of the weighting are, for example, the duration of participation and/or the ability to participate (‘stake’) measured by the network resources applied. The PoS thus can do without mining. In any case, the consensus algorithms enable each participant to check whether his or her stored blockchain complies with the rules of the whole.

3 For a direct transfer, for example, banks would no longer be necessary or notaries for the confirmation of contracts. In this way, corruption can be prevented, which makes the blockchain not only technologically economical but also socially relevant.

4 This is a protocol that allows scaling, especially of the Bitcoin blockchain, although the idea also seems to be applicable to other blockchains: here, the concept is that after a start signal, the so-called funding transaction, transactions made within a channel, are not stored in the blockchain. This results in a relief and thus potential scalability. After each payment, the current account balance is temporarily stored in the so-called commitment transaction and all transactions are only written to the blockchain when one of the participants closes the channel with a finalising signal, the so-called settlement transaction (Sixt 2017).

5 For example, in the public-permissionless Bitcoin blockchain, the validation time of a transaction can be up to two hours. In private-permissioned blockchains, it is usually only about 15 seconds (Baumann et al. 2017).
